# Inhibition of Retinoic Acid Receptor Gamma Improves Bovine Embryo Development

**DOI:** 10.3390/vetsci12100924

**Published:** 2025-09-24

**Authors:** Xiangyan Wang, Wenjing Wan, Yue Su, Shengcan Xie, Fenfen Jiang, Zhen Yang, Shuangyi Xiehe, Wei Ma, Linxiu Yue, Ningxiao Li, Ahui Wang, Jintong Guo, Xiaoting Li, Xinfeng Liu, Young Tang

**Affiliations:** 1 Key Laboratory of Livestock Biology, Shanxi Centre of Stem Cells Engineering & Technology, College of Veterinary Medicine, Northwest A&F University, Yangling 712100, China; wxy805841382@126.com (X.W.); wen-jingwan@nwafu.edu.cn (W.W.); sysuyue1993@163.com (Y.S.); xieshengcan@nwafu.edu.cn (S.X.); ffj@nwafu.edu.cn (F.J.); youngzhen@nwafu.edu.cn (Z.Y.); xhsy@nwafu.edu.cn (S.X.); mawei2000@nwafu.edu.cn (W.M.); ylx2021@nwafu.edu.cn (L.Y.); lningxiao@nwafu.edu.cn (N.L.); 2024060400@nwsuaf.edu.cn (A.W.); 2023050645@nwafu.edu.cn (J.G.); lixaoting@nwafu.edu.cn (X.L.); 2 Key Laboratory of Ministry of Education for Conservation and Utilization of Special Biological Resources in the Western, Ningxia University, Yinchuan 750021, China; liu2019074@nxu.edu.cn

**Keywords:** RARγ, bovine embryo, metabolic reprogramming

## Abstract

Retinoic acid receptor gamma (RARγ) plays a critical but poorly understood role in early embryo development and stem cell pluripotency. Here, we investigated the effects of the RARγ-specific inhibitor LY2955303 (LY) on in vitro-produced bovine embryos. Treatment with LY significantly increased blastocyst rates and quality, demonstrating its potential to enhance IVF outcomes in cattle. Single-embryo RNA sequencing using Smart-seq indicated that LY promotes metabolic reprogramming (upregulating glycolysis and TCA cycle activity) while suppressing apoptosis and inflammatory responses. LY treatment starting from the 16-cell stage led to enhanced glycolysis in blastocysts compared with exposure from the 2-cell stage. Furthermore, LY treatment also upregulated oxidative phosphorylation in bovine embryonic stem cells (ESCs). These findings establish RARγ as an important regulator of bovine embryo development, with its inhibition offering a novel strategy to optimize IVF embryo culture systems for livestock production.

## Simple Summary

Retinoic acid receptor gamma is important in early embryo development, but its exact role is unclear. Improving embryo quality during in vitro fertilization could benefit livestock breeding. We studied how the retinoic acid receptor gamma-specific inhibitor LY2955303 affects in vitro-fertilized cow embryos. LY increased the embryo blastocyst rate compared with the control. RNA-seq revealed that LY enhanced energy production while reducing cell death and inflammation. Starting LY at the 16-cell stage improved glycolysis more than earlier treatment. It also boosted energy metabolism in bovine embryonic stem cells. This study could lead to better livestock breeding methods, improving food production and farming efficiency.

## 1. Introduction

Retinoic acid (RA), a vitamin A metabolite, is essential for cell–cell signaling during vertebrate organogenesis [1] and governs embryonic patterning and differentiation [2,3]. It primarily acts through two families of nuclear receptors: retinoic acid receptors (RARs) and retinoid X receptors (RXRs). RAR consists of α, β, and γ subtypes (encoded by RARA/B/G, respectively), all of which can be activated by various forms of RA, unlike RXRs, which are activated exclusively by 9-cis RA [4,5]. In mice, RARγ is essential for zygotic genome activation (ZGA). Blocking RARγ with small chemical inhibitor LY2955303 (LY) arrested mouse embryo development at the two- to four-cell stages [6] and caused embryonic growth retardation [7], while it boosted the emergence of two-cell-like-cells from embryonic stem cells (ESCs) [6,8]. Additionally, RARγ inhibition suppresses the differentiation of esophageal epithelium and neuronal progenitors from human pluripotent stem cells (PSCs) [9,10]. However, species-specific differences exist: human preimplantation embryos lack RARγ expression during ZGA [11], indicating the complicated and context-dependent regulatory roles of RARs during evolution. Also, whether RAR activity plays a role in bovine embryogenesis is still uninvestigated.

Bovine in vitro fertilization (IVF) is pivotal and a transformative tool for livestock breeding. However, oocytes from slaughterhouse ovaries (SHOs) exhibit lower developmental competence than those from live donors from ovum pick-up (OPU) [12]. Today, more than 80% of bovine embryos are produced through IVF, with the actual number being over 2 million in 2022 [13]. The ability to produce embryos in vitro has several advantages, including the rapid genetic improvement of livestock herds, increased reproductive efficiency, and enhanced production of livestock with superior traits such as higher milk yield, disease resistance, and faster growth rates. It is therefore critical to further improve the success rates of early bovine IVF embryo development.

This study aimed to investigate the effect of the RARγ-specific inhibitor LY on bovine IVF embryos and the pluripotent PSCs derived from early bovine embryos–bovine embryonic stem cells (bESCs), to better understand the role of RARγ in bovine pluripotency and early embryo development. We investigated the effect of LY on the development of bovine embryos using SHO-derived oocytes. Contrary to the findings in mice, we found that the RARγ inhibitor improves bovine blastocyst rates and modulate the metabolism in bESCs.

## 2. Materials and Methods

### 2.1. Oocyte Collection and In Vitro Maturation (IVM)

The bovine ovaries, collected from a local slaughterhouse, were transported to the laboratory within 2–4 h in physiological saline containing penicillin/streptomycin at 37.0 °C. Upon arrival, the ovaries were promptly rinsed with 75% ethanol to minimize contamination risk, followed by thorough washing with physiological saline supplemented with 100 IU/mL penicillin/streptomycin to remove residual tissues. Ovarian follicles were incised with a sterile surgical blade to release cumulus–oocyte complexes (COCs), which were then collected under a microscope, selecting only those that were surrounded by at least three layers of compact cumulus cells. The selected COCs, exhibiting homogeneous cytoplasm and ≥3 cumulus cell layers, were randomly allocated into four groups and transferred to 4-well culture dishes containing 500 μL of maturation medium. Oocyte maturation was conducted for 22.5 h in a humidified incubator at 38.5 °C with 5% CO_2_. The maturation medium consisted of IVM (IVF Bioscience, 71001, Falmouth, UK) base supplemented with 20 ng/mL leukemia inhibitory factor (LIF; R&D Systems, 7734LF025, Minneapolis, MN, USA), 40 ng/mL fibroblast growth factor 2 (FGF2; Sigma, SRP4037, St. Louis, MO, USA), and 20 ng/mL insulin-like growth factor 1 (IGF1; R&D Systems, 291-G1, Minneapolis, MN, USA).

### 2.2. In Vitro Fertilization (IVF)

After maturation, oocytes were washed 2–3 times with IVF medium and transferred to 4-well dishes containing 400 μL of IVF (IVF Bioscience, 71004, Falmouth, UK) medium. Frozen bovine semen was thawed in a 38.0 °C water bath for 30 s, adjusted to a final concentration of 1 × 10^6^ sperm/mL, and introduced into the fertilization wells. Following 20 h of co-incubation, cumulus cells were removed by 1% hyaluronidase treatment (Sigma, H4272, St. Louis, MO, USA), and presumptive zygotes were transferred to IVC medium (IVF Bioscience, 71005, Falmouth, UK). On the following day, cleaved embryos were equally distributed into different experimental groups with the following IVC treatments: (1) DMSO control: IVC + 20 ng/mL LIF + 40 ng/mL FGF2 + 20 ng/mL IGF1 + 1:5000 DMSO; (2) LY 10 μM group: IVC + 20 ng/mL LIF + 40 ng/mL FGF2 + 20 ng/mL IGF1 + 10 μM LY2955303 (MCE, HY-107765, Montville, NJ, USA) (50 mM stock solution diluted 1:5000 in DMSO, matching the solvent concentration of the control); or (3) BMS-195614 10 μM group: IVC + 20 ng/mL LIF + 40 ng/mL FGF2 + 20 ng/mL IGF1 + 10 μM BMS-195614 (MCE, HY-101259, Montville, NJ, USA). Embryo development was assessed every 48 h until day 8.

### 2.3. Single-Embryo SMART-Seq

Transcriptome libraries from individual blastocysts were prepared using the SMART-Seq^®^ mRNA HT LP kit (Takara, 634794, Kyoto, Japan). Each blastocyst was lysed in 12.5 μL CDS Sorting Solution containing 10× Lysis Buffer, RNase Inhibitor, and 3′ SMART-Seq CDS Primer II A. cDNA synthesis was performed in a one-step reaction using SMART Scribe Reverse Transcriptase and SeqAmp DNA Polymerase (42 °C for 90 min, followed by 17–20 PCR cycles optimized for blastocyst samples). Amplified cDNA was purified using NucleoMag NGS Clean-up beads (Takara, 744970.5, Kyoto, Japan). Purified cDNA was enzymatically fragmented and ligated with stem-loop adapters. Indexed libraries were prepared using 12–16 PCR cycles with unique dual indexes (UDIs). Library quality was verified by TapeStation analysis (Agilent, G2992AA, Santa Clara, CA, USA) and quantified using Qubit dsDNA HS Assay (Thermo Fisher Scientific, Q33238, Waltham, MA, USA). Pooled libraries were sequenced on an Illumina NovaSeq 6000 (v1.5 chemistry) (2 × 150 bp). Raw reads were processed using FastQC for quality control, aligned to the reference genome using STAR, and quantified using feature Counts. Differential expression analysis was performed using DESeq2.

Raw data (raw sequencing reads) in a FASTQ format were initially processed using the fastp software (v0.23.2). Reference genome and gene model annotation files were obtained directly from genome databases. The reference genome index was constructed using HISAT2 v2.0.5, and paired-end clean reads were aligned to the reference genome using the same tool. Feature Counts v1.5.0-p3 was used to quantify reads mapped to each gene, and FPKM (Fragments Per Kilobase of transcript per Million mapped reads) was calculated based on gene length and mapped read counts. Subsequent RNA-seq data analysis was performed on the NovoMagic platform (https://magic-plus.novogene.com, accessed on 8 November 2024). Differential expression analysis between two conditions (with two to three biological replicates per group) was conducted using the DESeq2 R package (v1.20.0), with additional validation being performed using the edgeR R package (v3.22.5). Gene Ontology (GO) enrichment analysis and KEGG pathway analysis of differentially expressed genes (DEGs) were performed using the cluster Profiler R package (version 3.5.0), which corrects for gene length bias.

### 2.4. bESC Culture

The bovine embryonic stem cell (bESC) lines were derived from previous studies [14] and cultured according to established protocols. Briefly, bESCs were maintained on mitotically inactivated mouse embryonic fibroblast (MEF) feeder layers in TIFX culture medium. The TiFX medium comprised mTeSR-plus media (mTeSR Plus (STEMCELL, 100-0276, Cambridge, MA, USA), 1× penicillin–streptomycin (Gibco, 15140122, Glendale, CA, USA), 0.8 μM PD184352 (MEK1/2i; Selleck Chemicals, S1020, Houston, TX, USA), 2.5 μM IWR-1 (Selleck Chemicals, S7086, Houston, TX, USA), 3.3 μM EPZ004777 (DOT1Li; Selleck Chemicals, S7565, Houston, TX, USA), 2 μM SU5402, (Selleck Chemicals, S7667, Houston, TX, USA), 3 μM CHIR99021 (GSK3i; Selleck Chemicals, S2924, Houston, TX, USA), 10 μM Forskolin (Selleck Chemicals, S2449, Houston, TX, USA), and 1000 U/mL human LIF (R&D Systems, 7734LF025, Minneapolis, MN, USA). The cells were cultured at 37 °C in a humidified atmosphere containing 5% CO_2_. Upon reaching confluence, bESCs were passaged using TrypLE (Gibco, 12604021, Glendale, CA, USA) and replated onto fresh feeder layers.

### 2.5. Cell Metabolism Analysis

Cellular glycolytic rates were measured using the Agilent Seahorse XFp Glycolytic Rate Assay Kit (Agilent Technologies, 103346-100, Santa Clara, CA, USA). Cells were seeded in XFp Cell Culture Miniplates at optimized densities and equilibrated in XF DMEM medium (pH 7.4) supplemented with 10 mM glucose, 2 mM glutamine, and 1 mM pyruvate for 1 h in a non-CO_2_ incubator (37 °C). The sensor cartridge was hydrated overnight in XF Calibrant. The assay measured the extracellular acidification rate (ECAR) and oxygen consumption rate (OCR) in real time. Rotenone/Antimycin A (Rot/AA, 0.5 μM) was injected to inhibit mitochondrial respiration, followed by 2-deoxy-D-glucose (2-DG, 50 mM) to block glycolysis. Data were analyzed using the Seahorse Glycolytic Rate Assay Report Generator and calculating glycolytic proton efflux rate (glycoPER) by subtracting mitochondrial acidification from total proton efflux.

### 2.6. Statistical Analysis

All experiments were repeated three times, and SPSS version 17.0 was used for data analyses. Differences between the groups were analyzed with a *t* test. Values are expressed as the mean ± SEM, and a value of *p* < 0.05 was used to indicate statistical significance.

## 3. Results

### 3.1. LY2955303 Enhances Bovine IVF Embryo Development

**Figure 1:**
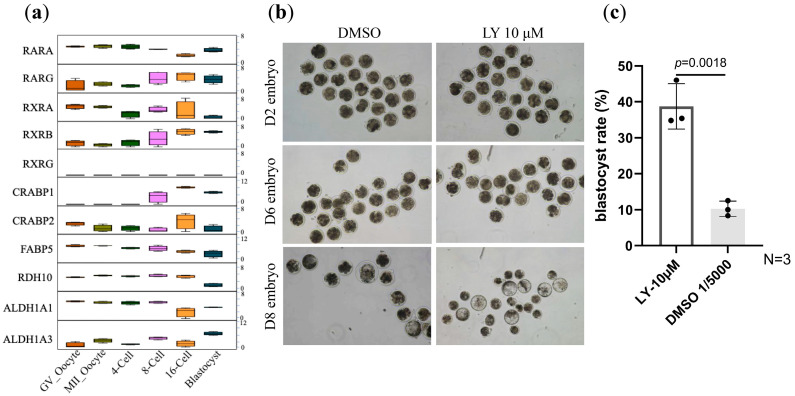
Expression of RA signaling-related genes and the effect of LY treatment on bovine embryo development. (**a**) Expression patterns of retinoic acid signaling-related genes across embryonic developmental stages. (**b**) Representative images of bovine embryos cultured under DMSO or 10 μM LY conditions (2-cell stage). (**c**) Quantification of blastocyst formation rates showing significantly enhanced embryonic development in LY-treated group (38.7%) compared to the DMSO control (10.3%) (*N* = 3 biological replicates; *p* = 0.0018, Student’s *t*-test). Data are presented as mean ± SEM.

The RARγ-specific inhibitor LY2955303 (LY) was reported to block ZGA development in mice, leading to development arrest at the two- to four-cell stages [6]. Unlike mice, bovine embryo minor and major ZGA happen at the four- and eight-cell stage, respectively. Through reanalyzing the previously reported RNAseq data [13], we determined that both RARA and RARG are expressed in oocytes and early embryos, with an obvious increase in RARG expression at the eight-cell stage (Figure 1a), indicating a stimulation of its activity during and after major ZGA. We therefore asked whether RARγ inhibition in bovine would cause an arrest of embryo development. Cumulous–oocyte complexes (COCs) were harvested from bovine ovaries collected from local slaughterhouse, and the oocytes matured. Standard in vitro fertilization (IVF) procedure was performed to these SHO-retrieved oocytes. Fertilized oocytes were collected, with an average cleavage rate of 74.2% at 48 h post IVF (Table S1), which is comparable to the previously reported cleavage rate for SHO-derived oocytes [13].

The cleaved oocytes (two-cell stage) were then randomly distributed into two groups, each cultured in a commercial in vitro culture (IVC) medium, with the addition of 10 μM LY as treatment and DMSO as the vehicle control. Blastocyst formation was evaluated 8 days after IVF. Interestingly, unlike what was found in mice, LY treatment (10 μM) did not arrest bovine embryo development but increased the blastocyst rate from 10.3% (DMSO) to 38.7% (Figure 1b,c, Table 1). However, inhibition of all RARs by using 10 μM BMS-195614 (a pan-antagonist of RAR*α*, *β*, and γ) did not have a significant impact on blastocyst formation (Figure S1A,B), indicating different regulatory effects by RAR*α*, *β* subtypes, on embryo development than by RARγ.

### 3.2. LY2955303 Treatment Alters mRNA Expression Profiles in Bovine Blastocysts

**Figure 2:**
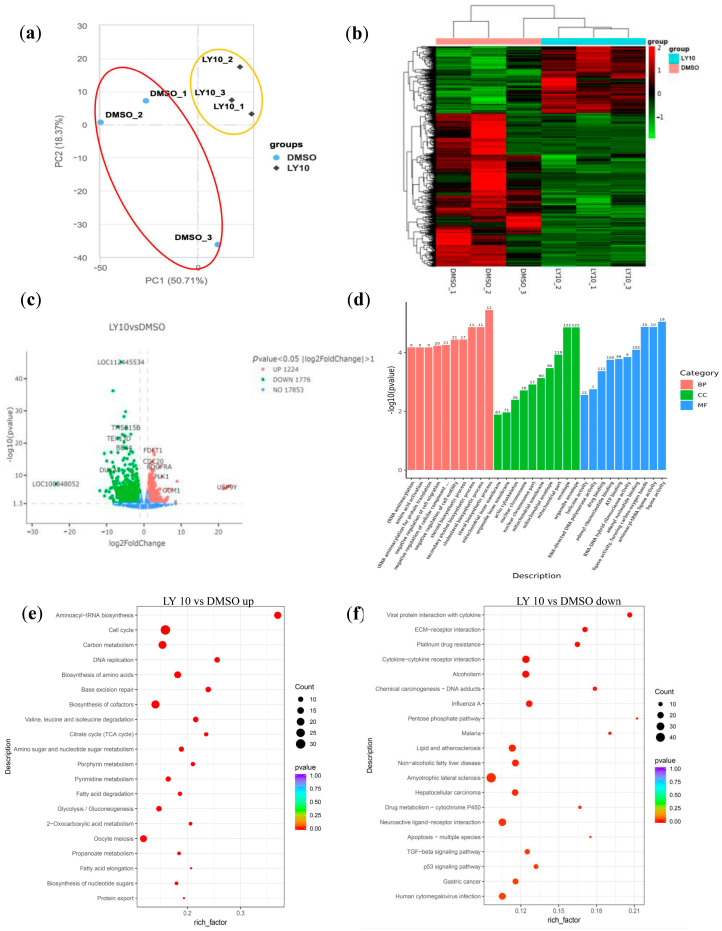
Transcriptomic profiling of bovine embryos treated with LY2955303 (LY). (**a**) PCA plot showing global gene expression differences (PC1: 50.71%; PC2: 18.37%). (**b**) Clustered heatmap of DEGs. The columns and rows in the heatmap represent samples and genes, respectively. Sample names are displayed below the heatmap. (**c**) Volcano plot of DEGs (|log2FC| > 1, *p* < 0.05; 1224 up, 1776 down). (**d**) The top 10 terms in biological processes (BPs), cellular components (CCs), and molecular functions (MFs) from the GO analysis. (**e**) KEGG pathway enrichment analysis of upregulated DEGs. (**f**) KEGG pathway enrichment analysis of downregulated DEGs.

To understand the potential mechanism through which LY improves bovine IVF embryo development, single-embryo SMART-seq was conducted for bovine IVF blastocysts under LY10 or DMSO conditions. Principal component analysis (PCA) grouped LY-treated bovine embryos separately from the DMSO control embryos (Figure 2a). A heatmap of the differentially expressed genes (DEGs) also clustered LY-treated embryos together (Figure 2b). Volcano plot analysis revealed that compared to the DMSO control group, the 10 μM LY treatment group exhibited 1224 significantly upregulated genes and 1776 significantly downregulated genes (Figure 2c). Gene Ontology (GO) enrichment analysis demonstrated that these DEGs were primarily enriched in biological processes (BPs) that are relevant to protein synthesis, such as tRNA aminoacetylation and amino acid activation, and in cellular components (CCs) that are relevant for mitochondria function (Figure 2d). Accordingly, Kyoto Encyclopedia of Genes and Genomes (KEGG) analysis of the upregulated DEGs further revealed that the LY-treated embryos are enriched with metabolic signaling events such as “Glycolysis/Gluconeogenesis”, “Citrate cycle (TCA cycle)”, “Pyruvate metabolism”, “Cell cycle”, etc. (Figure 2e, Table S2), indicating metabolic reprogramming of the embryonic cells to provide the necessary energy and biosynthetic precursors for rapid blastocyst formation. The enrichment of “Valine, leucine, and isoleucine degradation” and “propanoate metabolism” further supports enhanced amino acid catabolism, which may fuel the TCA cycle and support embryo growth. Also, terms like “Aminoacyl-tRNA biosynthesis”, “Biosynthesis of amino acids”, “Pyrimidine metabolism”, and “Nucleotide sugar biosynthesis”, etc., indicate enhanced protein and nucleic acid synthesis, which are crucial for rapid embryonic growth.

As for the downregulated DEGs, KEGG analysis of the LY-treated bovine embryos showed enrichment of terms associated with inflammatory and immune responses (Figure 2f, Table S3), including “Viral protein interaction with cytokine and cytokine receptor”, “Cytokine-cytokine receptor interaction”, and “Influenza A”. Also, LY-treated embryos showed decreases in stress and apoptosis pathways, such as “Apoptosis-multiple species”, “p53 signaling pathway”, and “TGF-beta signaling pathway”. Furthermore, the heatmap reveals that HOX family genes, direct targets of RAR stimulation for early embryo differentiation and patterning [15], were downregulated significantly after LY treatment (Figure S1C). Taking together, these results indicate that LY treatment improves the development potential of early bovine blastocysts, likely through improved energy metabolism and cell proliferation, as well as the suppression of inflammation, cell stress, and premature differentiation in early bovine embryos.

### 3.3. LY Promotes Post-ZGA Bovine Embryo Energy Metabolism

**Figure 3:**
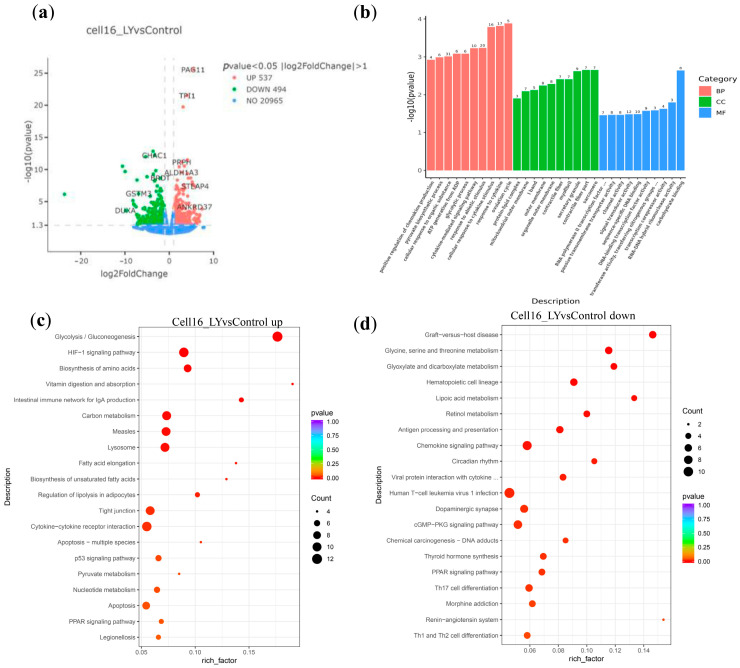
Transcriptomic profiling of bovine LY-treated blastocysts starting from the 16-cell stage versus DMSO control. (**a**) Volcano plot showing the differential gene expression between the LY-treated blastocysts from the 16-cell stage and the control group. (**b**) The top 10 terms in biological processes, cell components, and molecular functions from the GO analysis. (**c**) KEGG pathway enrichment analysis of upregulated differentially expressed genes. (**d**) KEGG pathway enrichment analysis of downregulated differentially expressed genes.

The RARG expression increases starting at ZGA in bovine embryos (Figure 1a, Table S4), indicating a potential regulatory role for morula and blastocyst stage development. We therefore asked if inhibiting RARγ activity by LY at the morula stage would affect the bovine embryo development. SMART-seq was performed to bovine blastocysts treated with LY from the 16-cell stage and the DMSO control. Treatment with LY resulted in 109 upregulated genes and 89 downregulated genes compared to the DMSO control group (Figure 3a). Similarly to LY-treated embryos from the two-cell stage (Figure 2d), these DEGs were functionally enriched in cellular components such as the sarcomere and mitochondrial outer membrane and were involved in biological processes, including the chemokine and cytokine response, and energy production such as glycolysis (Figure 3b). Accordingly, KEGG analysis on upregulated DEGs (Figure 3c) also revealed enrichment of metabolic activity and energy supply, such as “Glycolysis/Gluconeogenesis”, “Carbon metabolism”, “Pyruvate metabolism”, “Nucleotide metabolism”, and “Biosynthesis of amino acids”, etc., which is consistent with LY-treated embryos from the two-cell stage (Figure 2e). There is also upregulation of the “HIF-1 signaling pathway”, which regulates genes involved in glycolysis and cell survival. In addition, Lipid Metabolism and Membrane Formation processes are also present, such as “Fatty acid elongation”, “Biosynthesis of unsaturated fatty acids”, etc., indicating that RARγ inhibition promotes lipid droplet formation and energy reserves, which are essential for blastocyst expansion and implantation. However, there is also upregulation in “Apoptosis-multiple species” and “p53 signaling pathway” following LY treatment (Figure 3c, Table S5), which suggests increased cell death or, alternatively, a selective mechanism to eliminate damaged cells to ensure healthier blastocyst formation, which is yet to be verified.

For the downregulated DEGs, enrichment of KEGG terms by LY treatment indicates suppression of immune/inflammatory pathways, such as “Graft-versus-host disease”, “Antigen processing and presentation”, “Chemokine signaling”, etc. As expected, retinal metabolism is downregulated (Figure 3d, Table S6). Furthermore, the downregulated “Dopaminergic synapse”, “cGMP-PKG”, “Renin-angiotensin system”, etc., may indicate an inhibition of early neuronal differentiation signaling in preimplantation development.

**Figure 4:**
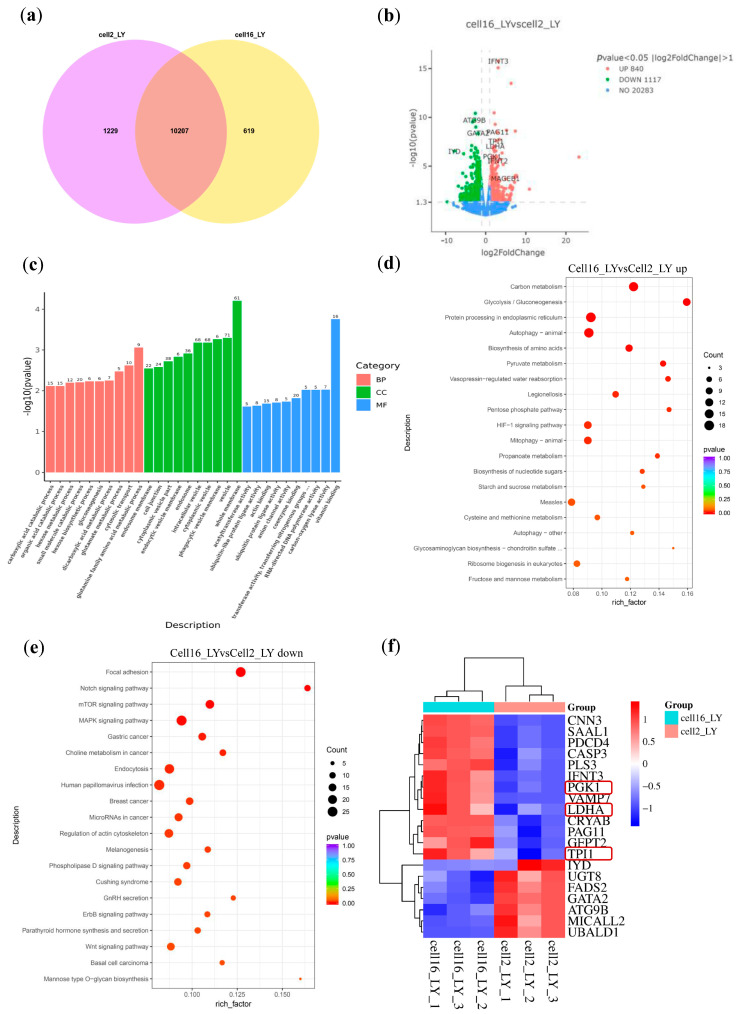
Transcriptomic analysis of bovine blastocysts treated with LY2955303 (LY) starting at the 16-cell and 2-cell stages. (**a**) Venn diagram showing the overlap of DEGs between cell16_LY and cell12_LY groups. (**b**) Volcano plot of DEGs in cell16_LY vs. cell12_LY comparison. Genes with *p*-value < 0.05 and |log_2_FoldChange| > 1 are categorized as upregulated (red, *N* = 840), downregulated (green, *N* = 1117), or non-significant (blue, *N* = 20,283). (**c**) GO enrichment analysis (BP: Biological Process; CC: Cellular Component; MF: Molecular Function) of DEGs, visualized as a bar plot showing −log_10_(*p*-value) for top enriched terms. (**d**) Bubble plot of KEGG pathway enrichment for upregulated DEGs in cell16_LY vs. cell12_LY. Dot size represents gene count; color indicates *p*-value. (**e**) Bubble plot of KEGG pathway enrichment for downregulated DEGs in cell16_LY vs. cell12_LY. Dot size represents gene count; color indicates *p*-value. (**f**) Heatmap of top DEGs (hierarchically clustered) distinguishing the cell16_LY and cell12_LY groups, showing normalized expression values (blue: low; red: high), the red boxes indicate the genes related to the glycolytic pathway.

We further questioned if there is a transcriptome difference between LY-treated blastocysts starting at the 16-cell and 2-cell stages. Venn diagram analysis revealed distinct gene expression patterns between these two treatments, with 619 and 1229 uniquely expressed genes for 16-cell and 2-cell treatment, respectively (Figure 4a). Volcano plot analysis demonstrated that LY treatment at the 16-cell stage specifically upregulated glycolysis-related genes, including TPI1, PGK1, and LDHA, compared to treatment at the 2-cell stage (Figure 4b). GO enrichment analysis indicated that these DEGs were primarily involved in various metabolic processes (Figure 4c). Interestingly, upregulated pathways also include those for energy production and biosynthesis, including “Carbon metabolism”, “Glycolysis”, “Pentose phosphate pathway”, “Pyruvate metabolism”, “Biosynthesis of amino acids/nucleotide sugars”, and “HIF-1 signaling pathway” (Figure 4d, Table S7). Also, multiple pathways are downregulated, such as “Notch, Wnt, MAPK, ErbB signaling”, indicating a delay of premature differentiation signaling (Figure 4e, Table S8). Subsequently, we performed hierarchical clustering of the top 20 differentially expressed genes, which again identified genes associated with glycolysis and HIF signaling pathways (Figure 4f). Taking together, these data indicate that LY treatment can upregulate energy metabolism in bovine post-ZGA-stage embryos.

### 3.4. LY Enhances Oxidative Phosphorylation in Bovine Embryonic Stem Cells

**Figure 5:**
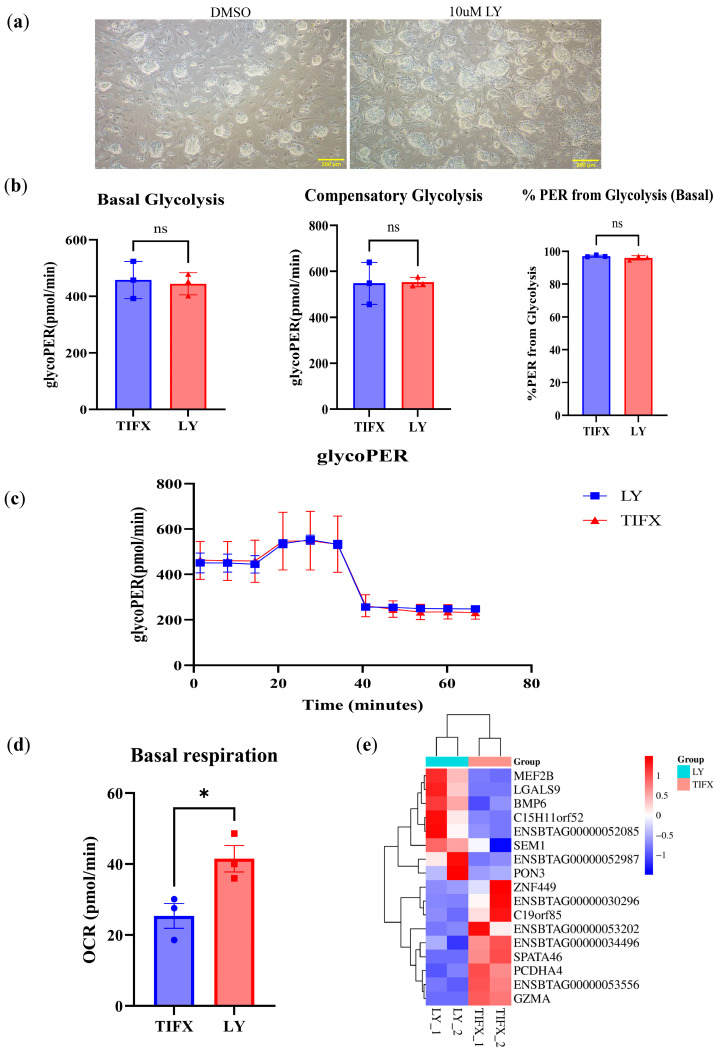
LY enhances oxidative phosphorylation in bovine embryonic stem cells. (**a**) Representative bright-field microscopy images of cells treated with DMSO (control) or 10 μM LY; scale bars = 200 μm; (**b**) glycolytic parameters analyzed by glycoPER assay: Basal Glycolysis, Compensatory Glycolysis, and the percentage of PER derived from glycolysis (Basal); (**c**) dynamic glycoPER (glycolytic proton efflux rate) profiles over an 80 min time course in TIFX (red)- and LY-treated (blue) cells; (**d**) basal respiration levels (OCR, oxygen consumption rate) measured in TIFX- and LY-treated cells; (**e**) hierarchical clustering heatmap of differentially expressed genes between LY (blue-labeled group) and TIFX (red-labeled group) treatment groups. The color scale represents log_2_-transformed fold change in gene expression, highlighting distinct transcriptional signatures associated with each treatment; * *p* < 0.05.

Preimplantation blastocysts contain two cell parts, the inner cell mass (ICM) as well as trophoblasts, which are the result of the first embryonic cell differentiation event. We recently reported the derivation of bovine embryonic stem cells (bESCs) from the ICM of Holstein cattle IVF blastocysts [14]. As LY treatment stimulated the energy metabolism in bovine embryos, we asked if LY treatment would also affect bESCs. Compared with the DMSO control, LY treatment of bESCs at 10 μM for 3 days led to smoother colony shape with a clearer edge (Figure 5a), although the cell growth rate was not significantly changed (Figure S1D).

To evaluate the impact of LY treatment on bESC metabolism, the extracellular acidification rate (ECAR) and oxygen consumption rates (OCRs) were measured using a Seahorse instrument. The ECAR and OCR revealed that the glycolysis rate is not affected by LY treatment in bESCs compared with the DMSO control (Figure 5b,c). However, the mitochondrial respiration rate increased significantly by LY treatment (Figure 5d). Transcriptomic analysis was performed on LY-treated and control bESCs. Among the limited number of DEGs that were identified in LY treatment versus the DMSO control (Figure 5e, Table S9), we identified the downregulation of GZMA and upregulation of MEF2B and LGALS9. GZMA upregulation triggers cell death by penetrating mitochondria to generate reactive oxygen species (ROS) and cleaves complex I protein to disrupt membrane potential [16], as well as by activating key pyroptosis protein GSDMB [17]. MEF2B can stimulate OXPHOS by activating mitochondria biogenesis genes [18], and LGALS9 is positively correlated with mitochondria activity [19]. These results indicate that LY inhibition enhances mitochondrial activity in bESCs, similarly to LY-treated blastocysts (Figure 2e).

## 4. Discussion

In mice, RA is critical during the totipotency window in early embryo development [6,7], and inhibition of RARγ with LY2955303 (LY) disrupts ZGA, leading to developmental arrest [6,7]. In bovines and humans, blocking endogenoud RA synthesis during the morula stage negatively interfered with embryo development, which could be reversed by the addition of RA [20,21], whereas exogenous RA alone did not affect the blastocyst rate but might improve embryonic cell proliferation, including ICM [20,21]. However, it remains unknown whether RARγ antagonism has a similar impact on early bovine embryo development and embryonic stem cells (ESCs). Our study reveals a striking divergence in the role of RARγ between mice and bovines: rather than impairing development, RARγ inhibition significantly enhances blastocyst formation in bovine IVF embryos. This finding underscores the importance of species-specific investigations in reproductive biology and challenges the assumption that RA signaling mechanisms are conserved across mammals.

The stage-dependent effects of LY treatment—administered at either the 2-cell or 16-cell stage—suggest that RARγ exerts distinct regulatory functions during bovine embryogenesis. The upregulation of metabolic pathways (e.g., glycolysis, TCA cycle) and proliferation-associated genes in LY-treated embryos aligns with the energy demands of rapid blastocyst formation. Notably, the suppression of inflammatory and stress-related pathways (e.g., cytokine signaling, p53 pathway) implies that RARγ inhibition creates a more favorable microenvironment for embryo development, potentially by reducing oxidative stress or immune-related cytotoxicity. This contrasts with mouse embryos, where RARγ blockade disrupts ZGA [6,7], highlighting a fundamental difference in how RA signaling coordinates early development between species.

The consistent upregulation of glycolysis and HIF-1 signaling in bovine embryos by LY treatment suggests that RARγ inhibition promotes a metabolic shift toward anaerobic energy production in embryos, which is advantageous for proliferating cells under in vitro conditions. This metabolic reprogramming mirrors the Warburg effect observed in cancer cells and pluripotent stem cells, where glycolysis supports biosynthetic precursors despite being less efficient than oxidative phosphorylation [22,23]. Our findings suggest that LY treatment may mimic a natural adaptation to hypoxia, a common challenge in early embryogenesis, thereby enhancing developmental competence. The blastocysts consist of two types of cells, the ICM and trophoblasts—the result of the first embryo differentiation. The fact that LY treatment enhanced mitochondrial respiration without sacrificing the glycolysis rate in bESCs suggest that RARγ may regulate cellular metabolism differently in TSCs than in ESCs and inhibit glycolysis in differentiated cells. Whether this caused the energy metabolism change observed in early embryos warrants further investigation. Also, the exact mechanism on how RARγ inhibition regulates metabolism (using ChIP-seq or metobolomics) warrants further investigation.

With over 2 million bovine embryos being produced annually via IVF [24], even modest improvements in blastocyst rates could have substantial economic and agricultural impacts. Our work identifies RARγ as a novel target for enhancing the efficiency of bovine IVF, particularly for oocytes from slaughterhouse-derived ovaries (SHOs), which typically exhibit lower developmental competence than OPU-derived oocytes [12]. By mitigating metabolic and inflammatory bottlenecks, LY treatment could democratize access to high-quality embryos, benefiting small-scale farms and global livestock industries alike.

In summary, we demonstrate that RARγ inhibition enhances bovine embryo development and ESC maintenance through metabolic reprogramming and suppression of stress pathways. These findings not only advance our understanding of species-specific RA signaling but also offer a translatable strategy to improve livestock reproductive technologies.

## Figures and Tables

**Table 1:** LY Promotes SHO oocyte IVF blastocyst rate compared with DMSO (*N* = 3).

Groups	Presumptive Zygotes in Culture	Blastocyst	Blastocyst Rate (%)
LY 10 μM	54	20	38.7 ± 7.3
DMSO	30	3	10.3 ± 2.2

## Data Availability

The original data presented in the study is available in the NCBI Geo Datasets (GSE305071).

## Supplementary Materials

The following supporting information can be downloaded at https://www.mdpi.com/article/10.3390/vetsci12100924/s1: Figure S1: Inhibition of retinoic acid receptor gamma improves bovine embryo development; Table S1: SHO oocyte IVF cleavage rate; Table S2: KEGG pathway enrichment analysis of upregulated genes in LY10 compared to DMSO; Table S3: KEGG pathway enrichment analysis of downregulated genes in LY10 compared to DMSO; Table S4: Fine mapping of genome activation in bovine embryos by RNA sequencing; Table S5: KEGG pathway enrichment analysis of upregulated genes in cell16_LY compared to control; Table S6: KEGG pathway enrichment analysis of downregulated genes in cell16_LY compared to control; Table S7: KEGG pathway enrichment analysis of upregulated genes in cell16_LY compared to cell2_LY; Table S8: KEGG pathway enrichment analysis of downregulated genes in cell16_LY compared to cell2_LY; Table S9: Differentially expressed genes between stem cell LY-treated group and DMSO control group.

## Abbreviations

The following abbreviations are used in this manuscript:

RA	retinoic acid
RARs	retinoic acid receptors
RXRs	retinoid X receptors
ZGA	zygotic genome activation
ESCs	embryonic stem cells
PSCs	pluripotent stem cells
IVF	in vitro fertilization
SHO	slaughterhouse ovaries
OPU	ovum pick-up
IVM	in vitro maturation
COCs	cumulus–oocyte complexes

## References

[1] Duester G. (2008). Retinoic acid synthesis and signaling during early organogenesis. Cell.

[2] Cunningham T.J., Duester G. (2015). Mechanisms of retinoic acid signalling and its roles in organ and limb development. Nat. Rev. Mol. Cell Biol..

[3] Rhinn M., Dolle P. (2012). Retinoic acid signalling during development. Development.

[4] Allenby G., Bocquel M.T., Saunders M., Kazmer S., Speck J., Rosenberger M., Lovey A., Kastner P., Grippo J.F., Chambon P. (1993). Retinoic acid receptors and retinoid X receptors: Interactions with endogenous retinoic acids. Proc. Natl. Acad. Sci. USA.

[5] Egea P.F., Rochel N., Birck C., Vachette P., Timmins P.A., Moras D. (2001). Effects of ligand binding on the association properties and conformation in solution of retinoic acid receptors RXR and RAR. J. Mol. Biol..

[6] Iturbide A., Ruiz Tejada Segura M.L., Noll C., Schorpp K., Rothenaigner I., Ruiz-Morales E.R., Lubatti G., Agami A., Hadian K., Scialdone A. (2021). Retinoic acid signaling is critical during the totipotency window in early mammalian development. Nat. Struct. Mol. Biol..

[7] Xu Y., Zhao J., Ren Y., Wang X., Lyu Y., Xie B., Sun Y., Yuan X., Liu H., Yang W. (2022). Derivation of totipotent-like stem cells with blastocyst-like structure forming potential. Cell Res..

[8] Gao R., Yang G., Wang M., Xiao J., Yi S., Huang Y., Guo Z., Kang Y., Fu Q., Wang M. (2024). Defining a TFAP2C-centered transcription factor network during murine peri-implantation. Dev. Cell.

[9] Das M., Pethe P. (2021). Differential expression of retinoic acid alpha and beta receptors in neuronal progenitors generated from human embryonic stem cells in response to TTNPB (a retinoic acid mimetic). Differentiation.

[10] Koterazawa Y., Koyanagi-Aoi M., Uehara K., Kakeji Y., Aoi T. (2020). Retinoic acid receptor γ activation promotes differentiation of human induced pluripotent stem cells into esophageal epithelium. J. Gastroenterol..

[11] Wu J., Xu J., Liu B., Yao G., Wang P., Lin Z., Huang B., Wang X., Li T., Shi S. (2018). Chromatin analysis in human early development reveals epigenetic transition during ZGA. Nature.

[12] Landeo L., Zuniga M., Gastelu T., Artica M., Ruiz J., Silva M., Ratto M.H. (2022). Oocyte Quality, In Vitro Fertilization and Embryo Development of Alpaca Oocytes Collected by Ultrasound-Guided Follicular Aspiration or from Slaughterhouse Ovaries. Animals.

[13] Graf A., Krebs S., Zakhartchenko V., Schwalb B., Blum H., Wolf E. (2014). Fine mapping of genome activation in bovine embryos by RNA sequencing. Proc. Natl. Acad. Sci. USA.

[14] Li N., Yang Z., Su Y., Ma W., Zhao J., Wang X., Wan W., Xie S., Li H., Wang M. (2025). Establishing Bovine Embryonic Stem Cells and Dissecting Their Self-Renewal Mechanisms. Int. J. Mol. Sci..

[15] Nolte C., De Kumar B., Krumlauf R. (2019). Hox genes: Downstream “effectors” of retinoic acid signaling in vertebrate embryogenesis. Genesis.

[16] Martinvalet D., Dykxhoorn D.M., Ferrini R., Lieberman J. (2008). Granzyme A cleaves a mitochondrial complex I protein to initiate caspase-independent cell death. Cell.

[17] Zhou Z., He H., Wang K., Shi X., Wang Y., Su Y., Wang Y., Li D., Liu W., Zhang Y. (2020). Granzyme A from cytotoxic lymphocytes cleaves GSDMB to trigger pyroptosis in target cells. Science.

[18] Hoang N.M., Liu Y., Bates P.D., Heaton A.R., Lopez A.F., Liu P., Zhu F., Chen R., Kondapelli A., Zhang X. (2024). Targeting DNMT3A-mediated oxidative phosphorylation to overcome ibrutinib resistance in mantle cell lymphoma. Cell Rep. Med..

[19] Sakhnevych S.S., Yasinska I.M., Fasler-Kan E., Sumbayev V.V. (2019). Mitochondrial Defunctionalization Supresses Tim-3-Galectin-9 Secretory Pathway in Human Colorectal Cancer Cells and Thus Can Possibly Affect Tumor Immune Escape. Front. Pharmacol..

[20] Gomez E., Caamano J.N., Rodriguez A., De Frutos C., Facal N., Diez C. (2006). Bovine early embryonic development and vitamin A. Reprod. Domest. Anim..

[21] Rodriguez A., Diez C., Ikeda S., Royo L.J., Caamano J.N., Alonso-Montes C., Goyache F., Alvarez I., Facal N., Gomez E. (2006). Retinoids during the in vitro transition from bovine morula to blastocyst. Hum. Reprod..

[22] Hsu P.P., Sabatini D.M. (2008). Cancer cell metabolism: Warburg and beyond. Cell.

[23] Cha Y., Han M.J., Cha H.J., Zoldan J., Burkart A., Jung J.H., Jang Y., Kim C.H., Jeong H.C., Kim B.G. (2017). Metabolic control of primed human pluripotent stem cell fate and function by the miR-200c-SIRT2 axis. Nat. Cell Biol..

[24] Krisher R.L., Herrick J.R. (2024). Bovine embryo production in vitro: Evolution of culture media and commercial perspectives. Anim. Reprod..

