# Supplementary material for: Inhibition of Retinoic Acid Receptor Gamma Improves Bovine Embryo Development
Source: Vet Sci. 2025 Sep 24;12(10):924. doi: 10.3390/vetsci12100924 (PMC12567714; doi:10.3390/vetsci12100924)
Supplement: Supplementary file 1 [file vetsci-12-00924-s001.zip › Supplementary Figure S1.pdf]

Supplementary Figure

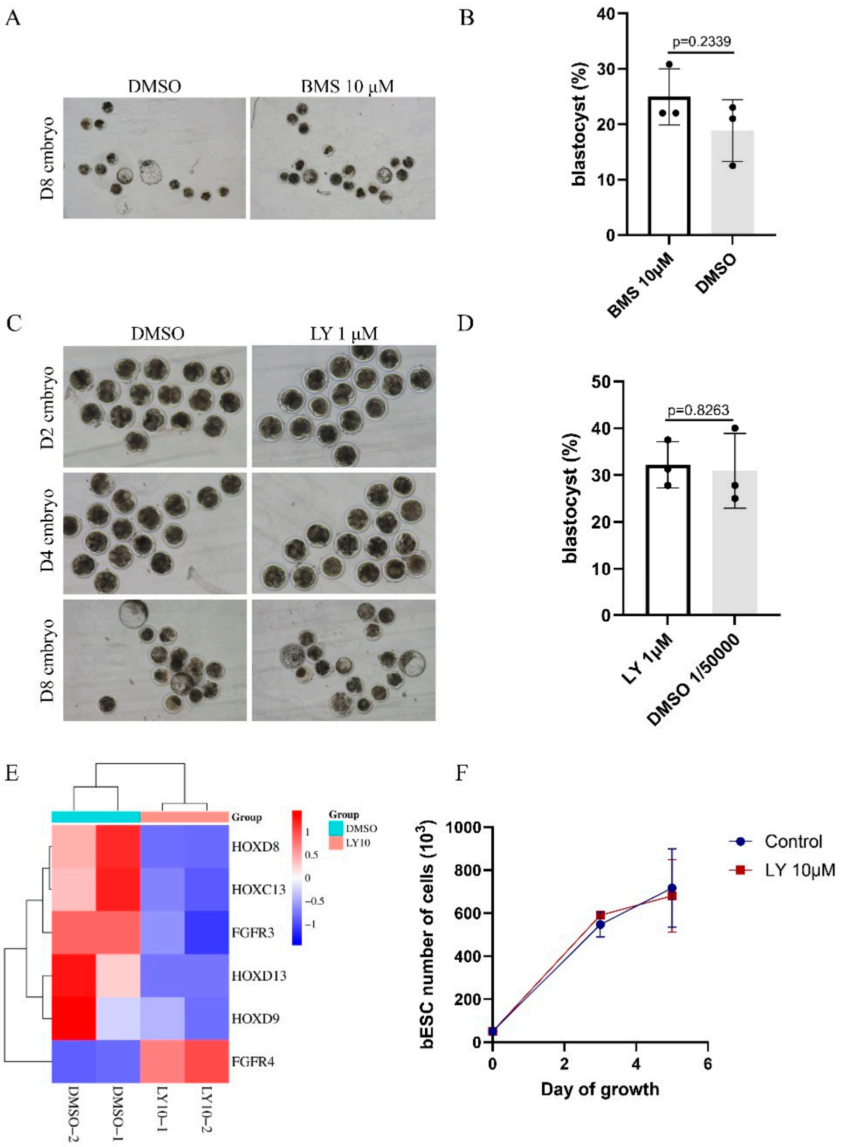

Figure S1: Inhibition of Retinoic Acid Receptor Gamma Improves Bovine Embryo Development. (A) Representative images of bovine embryos cultured under DMSO or 10 µM BMS-195614 conditions (2-cell stage). (B) Statistics of blastocyst rate after treating embryos with 10 µM BMS-195614 and DMSO. (C) Representative images of bovine embryos cultured under DMSO or 1 µM LY conditions (2-cell stage). (D) Statistics of blastocyst rate after treating embryos with 1 µM LY and DMSO. (E) Heatmap of the expression of RA-related target genes in different treatment groups. (F) Growth curves of bESCs under different conditions.(N=3 biological replicates, Student's t-test). Data are presented as mean ± SEM.
